# Nose-Only Exposure to Cherry- and Tobacco-Flavored E-Cigarettes Induced Lung Inflammation in Mice in a Sex-Dependent Manner

**DOI:** 10.3390/toxics10080471

**Published:** 2022-08-13

**Authors:** Thomas Lamb, Thivanka Muthumalage, Jiries Meehan-Atrash, Irfan Rahman

**Affiliations:** Department of Environmental Medicine, School of Medicine & Dentistry, University of Rochester Medical Center, Rochester, NY 14620, USA

**Keywords:** e-cigarettes, ENDS, flavors, tobacco, mint, lung, inflammation

## Abstract

Flavoring chemicals in electronic nicotine delivery systems have been shown to cause cellular inflammation; meanwhile, the effects of fruit and tobacco flavors on lung inflammation by nose-only exposures to mice are relatively unknown. We hypothesized that exposure to flavored e-cigarettes would cause lung inflammation in C57BL/6 J mice. The mice were exposed to air, propylene glycol/vegetable glycerin, and flavored e-liquids: Apple, Cherry, Strawberry, Wintergreen, and Smooth & Mild Tobacco, one hour per day for three days. Quantification of flavoring chemicals by proton nuclear magnetic resonance spectroscopy (^1^H NMR), differential cell counts by flow cytometry, pro-inflammatory cytokines/chemokines by ELISA, and matrix metalloproteinase levels by western blot were performed. Exposure to PG/VG increased neutrophil cell count in lung bronchoalveolar lavage fluid (BALF). KC and IL6 levels were increased by PG/VG exposure and female mice exposed to Cherry flavored e-cigarettes, in lung homogenate. Mice exposed to PG/VG, Apple, Cherry, and Wintergreen increased MMP2 levels. Our results revealed flavor- and sex-based e-cigarette effects in female mice exposed to cherry-flavored e-liquids and male mice exposed to tobacco-flavored e-liquids, namely, increased lung inflammation.

## 1. Introduction

Electronic nicotine delivery systems (ENDS), also referred to as electronic cigarettes (e-cigarettes), are devices that utilize an atomizer to aerosolize a liquid typically composed of propylene glycol/vegetable glycerin (PG/VG), nicotine, and flavoring chemicals at various concentrations [1]. In 2018, the United States had more than 8000 flavors and 250 e-cigarette brands available on the market [2]. In 2018, an estimated 8 million US adults (3.2%) were active e-cigarette users, with a high prevalence in young adults, with active e-cigarette users increasing to 4.5% in 2019 [3,4].

A majority of e-cigarette users list available flavor choices as their reason for initiation [5]. The Population Assessment of Tobacco and Health Study found age-dependent flavor preferences: adolescents have a higher affinity for fruit flavors than adults (52.8% vs. 30.8%), but a decreased preference for both menthol/mint (10.8% vs. 17.9%) and tobacco (5.1% vs. 24.5%) [6]. The 2021 National Youth Tobacco Survey also found fruit to be the preferred flavor among middle and high school students (71.6%), with mint and menthol trailing at 30.2% and 28.8%, respectively [7].

In the United States, current e-cigarette users believe that e-cigarettes are less harmful than traditional cigarettes [8,9]. Despite the fact that many flavoring chemicals are generally recognized as safe for ingestion (GRAS), emerging literature indicates that these chemicals may pose health risks to e-cigarette users [2]. A recent study demonstrated that ethyl maltol, maltol, ethyl vanillin, and furaneol exhibit cytotoxicity towards lung epithelial cells and mouse neuronal stem cells at concentrations found in e-liquids [10]. Monocytes treated with maltol, o-vanillin, and coumarin, and lung epithelial cells treated with maltol, o-vanillin, and diacetyl released significantly elevated levels of IL-8 [2,11,12]. Flavoring chemicals such as maltol and o-vanillin have been found in both fruit- and tobacco-flavored e-liquids [13]. Additionally, treatments with cinnamaldehyde-containing e-liquids decreased the phagocytotic activity of macrophages and neutrophils with concomitant increases in pro-inflammatory cytokine/chemokine secretion in the latter [14]. Recent studies also indicate that e-cigarette use is also beginning to be associated with lung remodeling and fibrosis-like events along with an increased risk for the development of respiratory diseases [15,16,17,18].

Given the high preference of flavored e-cigarette use in current users and in vitro data showing the induction of an inflammatory response by flavoring chemicals used in e-cigarettes, we hypothesize that nose-only exposure of mice to flavored e-cigarettes would result in lung inflammation. To conduct this study, we exposed mice to five different e-cigarette flavors to several puffs daily, a similar number to the daily puffs of e-cigarette users, by utilizing a puffing profile that mimicked the puffing topography of current e-cigarette users and measured pro-inflammatory cytokine levels, BALF cell counts, and lung protease levels to determine lung inflammation [19].

## 2. Materials and Methods

### 2.1. Ethics Statement

Experiments were performed following the standards established by the United States Animal Welfare Act. The Animal Research Committee of the University of Rochester (UCAR) approved the animal experimental protocol.

### 2.2. Animals

Six-week-old male and female C57 BL/6 J mice were ordered from Jackson Laboratory. Mice were housed at the University of Rochester for 1 week to acclimatize prior to nose-only tower training. All mice, regardless of exposure group, were trained by placing each mouse in the restraints of the Scireq nose-only tower one week prior to e-cigarette exposure. Mice were trained for fifteen minutes on the first day, thirty minutes on the second day, forty-five minutes on the third day, and one hour on the fourth and fifth days.

### 2.3. E-Cigarette Device and Liquids

A Joytech eVIC mini device (SCIREQ, Montreal) with KangerTech 0.15 Ω atomizers/coils (SCIREQ, Montreal) and the Scireq nose-only tower (SCIREQ, Montreal) were utilized for all e-cigarette exposures. E-liquids (0 mg nicotine), PG, and VG were purchased from the same company through local vendors/online vendors with e-liquids purchased under the following flavor categories, fruit (Apple, Cherry, and Strawberry), mint/menthol (Wintergreen), and tobacco (Smooth & Mild Tobacco). A 1:1 PG/VG mixture was used for all experiments.

### 2.4. E-Cigarette Exposure

E-cigarette nose-only exposure was performed utilizing the Scireq InExpose system using the Joytech eVIC mini device controlled by the Scireq Flexiware software. The puffing profile utilized to expose mice was set at two puffs per minute at an inter-puff interval of thirty seconds, with a three-second puff duration and a puff volume of 51 mL. Mice were split into six exposure groups (PG/VG, Apple, Cherry, Strawberry, Wintergreen, Smooth & Mild Tobacco) of equal numbers of males (3) and females (3) and exposed using the puffing profile (120 puffs daily) for a total of one hour per day for a three-day exposure. Air mice were exposed to room air following the same exposure methodology.

### 2.5. BALF Collection and Cell Counts

Mice were sacrificed 24 h after the last e-cigarette exposure and were euthanized by administering a mixture of ketamine and xylazine. Lungs were lavaged via tracheal catheterization three times each with 0.6 mL of 0.05% fetal bovine serum in 0.9% NaCl. The combined lavage fluids were centrifuged at 2000 rpm for 10 min at 4 °C. The supernatant was recovered and stored at −80 °C, while the cell pellet was resuspended in 1 mL of 1 × phosphate buffer saline (PBS). Total cell counts were measured by staining cells with acridine orange and propidium iodide (AO/PI) and counted using the Nexcelom Cellometer Auto 2000 cell viability counter. Differential cell counts were determined by flow cytometry using a Guava easyCyte flow cytometer with a minimum of 100,000 cells per sample. Cells from BALF were stained with CD16/32 (Tonbo biosciences 70-0161-u500, 1:10) to block nonspecific binding and then cells were stained using a master mix of CD45.1 (Biolegend Cat# 110728, 1:1000, San Diego, CA, USA), F4/80 (Biolegend Cat# 123110, 1:500, San Diego, CA, USA), Ly6 B.2 (Novus Biological Cat# NBP2-13077, 1:250, Littleton, CO, USA), CD4 (Invitrogen Cat# 25-0041-82, 1:500, Carlsbad, CA, USA), and CD8 (Invitrogen Cat# 17-0081-82, 1:500, Carlsbad, CA, USA).

### 2.6. Protein Extraction

Mouse lung lobes were collected and washed in 1 × PBS, dry blotted using a filter pad, and stored at −80 °C. Approximately 30 mg of lung tissue were mechanically homogenized in 350 μL of radioimmunoprecipitation assay buffer containing protease inhibitor and EDTA. After mechanical homogenization, samples were placed on ice for forty-five minutes before centrifugation at 14,000 rpm for thirty minutes at 4 °C. The supernatant was collected and stored at −80 °C in 50 μL aliquots for ELISA and Western blot. To determine the total protein concentration in each sample, the Pierce BCA Protein Assay kit (ThermoFisher Scientific, Cat# 23225, Waltham, MA, USA) was used and bovine serum albumin was utilized as the protein standard.

### 2.7. Pro-Inflammatory Cytokines/Chemokines

Pro-inflammatory cytokine/chemokine keratinocytes-derived chemokine (KC) (R&D DuoSet DY453), interleukin-6 (IL-6) (R&D Duoset DY406), and monocyte chemoattractant protein-1 (MCP-1) (R&D DuoSet DY479) levels were measured using ELISA following manufacturer protocol in BALF and lung homogenate. A dilution of 1:10 was utilized for lung homogenate samples and no dilution was utilized for BALF samples.

### 2.8. Immunoblot Assay

Equal concentration of lung homogenate samples, 10 μg of samples, were loaded per well of a 26 well 4–15% Criterion Precast Protein Gel (BioRad Cat# 5671085, GmbH, Feldkirchen, Germany) and proteins were ran at 200 V through the gel before being transferred to a nitrocellulose membrane. Nonspecific binding was blocked by incubating membranes in 5% non-fat milk in 1 × tris-buffer saline with 0.1% tween 20 (TBST) for one hour with rocking at room temperature. Membranes were then probed to determine protein levels using the following antibodies diluted in 5% non-fat milk in 1 × TBST: matrix metalloproteinase 9 (MMP9) (Abcam ab38898, 1:1000, Cambridge, MA, USA) and MMP2 (Abcam ab92536, 1:1000, Cambridge, MA, USA) and left rocking overnight at 4 °C. After overnight incubation, membranes were washed three times with 1 × TBST for ten minutes per wash and then incubated, with a secondary goat anti-rabbit antibody (BioRad Cat# 1706515, 1:10,000, GmbH, Feldkirchen, Germany) for one hour with rocking at room temperature. Membranes were then washed three times with 1 × TBST for ten minutes per wash and signals were measured using an ultra-sensitive enhanced chemiluminescent (Thermofisher Cat# 34096, Waltham, MA, USA) following the manufacturer’s protocol. Images of the membrane were collected utilizing the Bio-Rad ChemiDoc MP Imaging system (Bio-Rad Laboratories, GmbH, Feldkirchen, Germany). Membranes were then stripped utilizing restore western stripping buffer (Thermofisher Cat# 21063, Waltham, MA, USA) and re-probed for the other MMP and finally for β-actin (cell signaling 12620 s, 1:2000). Band intensity was determined using densitometry analysis using image lab software and normalized to the levels of β-actin. Fold changes in protein levels were relative to the protein levels of air-exposed mice.

### 2.9. Proton Nuclear Magnetic Resonance Spectroscopy Chemical Assay

In total, 120 μL e-liquids, 600 μL of DMSO-d6 containing 0.3% tetramethylsilane (Cambridge Isotope Laboratories Inc., Cat#DLM-10 TC-25, Andover, MA, USA), and 10 μL of a 306 mM solution of 1,2,4,5-tetrachloro-3-nitrobenzene in DMSO-d6 were combined, after which 500 μL of this mixture was introduced into 5 mm Wilmad 528-PP-7 thin wall precision NMR tubes for analysis. ^1^H NMR spectra were acquired on a Bruker Avance 500 MHz NMR spectrometer with 128 scans with a 4.7 s repetition rate, a 30° flip angle, with 64 k data points. Spectra were processed using Mestrenova with 0.3 Hz line-broadening factor to a final data size of 64 k real data points, manually phase-corrected, and baseline corrected using the Bernstein polynomial fit. Flavoring chemical concentrations were determined by comparing the peak integrations of the internal standard to flavoring chemicals, and the PG/VG ratio was determined by direct integration of their resonances. All samples were run in triplicates.

### 2.10. Statistical Analysis

Analysis was performed using GraphPad Prisma version 8.1.1 (San Diego, CA, USA) utilizing a one-way ANOVA with Dunnett’s multiple comparisons test with data shown as mean ± standard error of the mean (SEM).

## 3. Results

### 3.1. NMR Analysis of Flavored E-liquids for Flavoring Chemicals

The chemical composition of all e-liquids was assessed by NMR to determine the ratio of PG to VG and quantify key flavoring chemicals in flavored e-liquids. In the Apple e-liquid, the concentration of hexyl acetate was determined to be 0.43 ± 0.04 mg/mL, and ethyl maltol was determined to be 0.30 ± 0.05 mg/mL with a 46:54 PG/VG ratio (Table 1). In the Cherry e-liquid, the concentration of benzaldehyde was determined to be 0.12 ± 0.01 mg/mL with a 51:49 PG/VG ratio (Table 1). In the Strawberry e-liquid, the concentration of ethyl maltol was determined to be 0.32 ± 0.05 mg/mL and maltol was determined to be 0.24 ± 0.04 mg/mL with a 50:50 PG/VG ratio (Table 1). In the Wintergreen e-liquid, the concentration of methyl salicylate was determined to be 9.70 ± 0.50 mg/mL with a 49:51 PG/VG ratio (Table 1). Finally, in the Smooth & Mild Tobacco e-liquid, the concentration of maltol was determined to be 1.13 ± 0.02 mg/mL with a 49:51 PG/VG ratio (Table 1).

### 3.2. Alterations in Inflammatory Cell Influx in Lung by E-cigarette Flavors

**Figure 1:**
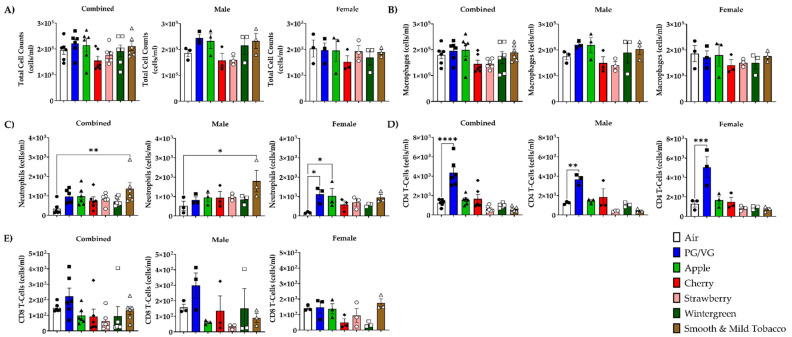
Sex-dependent effects of flavored e-cigarette exposure on inflammatory cell count in bronchoalveolar lavage fluid. Mice were exposed to air, PG/VG, and e-liquid flavors “Apple”, “Cherry”, “Strawberry”, “Wintergreen”, and “Smooth & Mild Tobacco” for 3 days for 1 h per day. Mice were sacrificed twenty-four hours after the final exposure. (**A**) Total cell counts were obtained by staining cells with AO/PI and counting with a cellometer. Differential cells were measured using flow cytometry: (**B**) F4/80+ macrophages, (**C**) Ly6 B.2+ neutrophils, (**D**) CD4+ T-cells, and (**E**) CD8+ T-cells. Data are shown as mean ± SEM with individual data points represented by the following symbols: Air (black circles), PG/VG (black squares), Apple (black triangles), Cherry (black diamonds), Strawberry (white circles), Wintergreen (white squares), Smooth & Mild Tobacco (white triangles), with * indicating *p* < 0.05, ** *p* < 0.01, *** *p* < 0.001, and **** *p* < 0.0001 vs. air controls. *n* = 6 for combined groups and *n* = 3 for male- and female-only groups.

To determine the effect of flavored e-cigarettes on the influx of inflammatory cells, differential cell counts were measured in BALF cells. In all mouse e-cigarette exposure groups, there were no significant alterations in total cell counts or macrophage cell counts compared to air controls (Figure 1A,B). In combined data, mice exposed to Smooth & Mild Tobacco resulted in a significant increase in the neutrophil cell count compared to air controls (Figure 1C). In male mice, exposure to Smooth & Mild Tobacco resulted in a significant increase in neutrophil cell counts, and in female mice, exposure to PG/VG and Apple resulted in a significant increase in neutrophil cell count compared to air controls (Figure 1C). Mice exposed to PG/VG resulted in a significant increase in the CD4 T-cell count compared to air controls (Figure 1D). In all mouse e-cigarette exposure groups, there were no significant alterations in CD8 T-cell count compared to air controls (Figure 1E).

### 3.3. Alteration of Pro-Inflammatory Cytokines/Chemokines Levels in Lungs by E-cigarette Flavors

**Figure 2:**
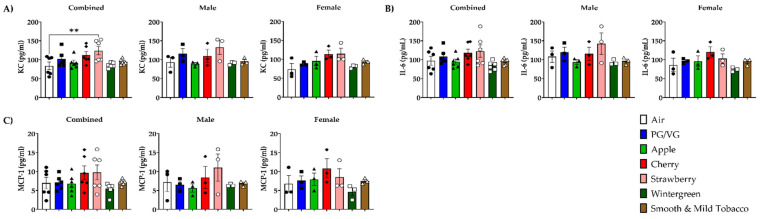
Sex-dependent effects of flavored e-cigarette exposure on pro-inflammatory cytokines/chemokine release in bronchoalveolar lavage fluid. Mice were exposed to air, PG/VG, and e-liquid flavors “Apple”, “Cherry”, “Strawberry”, “Wintergreen”, and “Smooth & Mild Tobacco” for 3 days for 1 h per day. Mice were sacrificed twenty-four hours after the final exposure. Pro-inflammatory cytokines/chemokines were measured in BALF. (**A**) KC levels, (**B**) IL-6 levels, (**C**) MCP-1 levels. Data are shown as mean ± SEM with individual data points represented by the following symbols: Air (black circles), PG/VG (black squares), Apple (black triangles), Cherry (black diamonds), Strawberry (white circles), Wintergreen (white squares), Smooth & Mild Tobacco (white triangles), with ** *p* < 0.01 vs. air controls. *n* = 6 for combined groups and *n* = 3 for male- and female-only groups.

**Figure 3:**
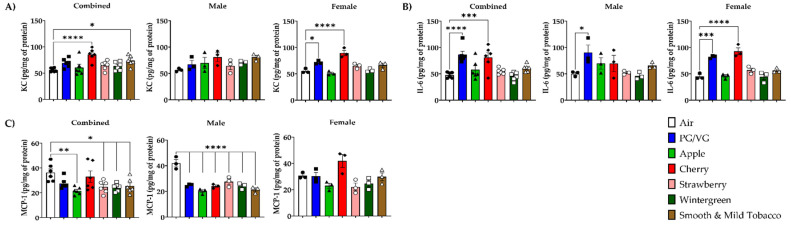
Sex-dependent effects of acute flavored e-cigarette exposure on pro-inflammatory cytokines/chemokine release in lung homogenate. Mice were exposed to air, PG/VG, and e-liquid flavors “Apple”, “Cherry”, “Strawberry”, “Wintergreen”, and “Smooth & Mild Tobacco” for 3 days for 1 h per day. Mice were sacrificed twenty-four hours after the final exposure. Pro-inflammatory cytokines/chemokines were measured in lung homogenate. (**A**) KC levels, (**B**) IL-6 levels, (**C**) MCP-1 levels. Data are shown as mean ± SEM with individual data points represented by the following symbols: Air (black circles), PG/VG (black squares), Apple (black triangles), Cherry (black diamonds), Strawberry (white circles), Wintergreen (white squares), Smooth & Mild Tobacco (white triangles), with * *p* < 0.05, ** *p* < 0.01, *** *p* < 0.001, and **** *p* < 0.0001 vs. air controls. *n* = 6 for combined groups and *n* = 3 for male- and female-only groups.

To determine the potential for flavored e-cigarette to elicit an inflammatory response, pro-inflammatory cytokines/chemokines were measured in BALF and lung homogenate. In BALF, KC levels in combined data were significantly increased in Strawberry-exposed mice compared to air controls (Figure 2A). In lung homogenate, KC levels in combined data were significantly increased in Cherry and Smooth & Mild Tobacco exposed mice compared to air controls (Figure 3A). In lung homogenate, there was no significant change in any exposed groups in male mice, but in female mice, there was a significant increase in KC levels when exposed to PG/VG and Cherry compared to air controls (Figure 3A). In BALF, IL-6 levels in all exposed mice were not significantly changed compared to air controls (Figure 2B). In lung homogenate, IL-6 levels in combined data were significantly increased in PG/VG and Cherry exposed mice compared to air controls (Figure 3B). In lung homogenate, there was a significant increase in IL-6 levels in male mice exposed to PG/VG as compared to air controls, and female mice exposed to PG/VG and Cherry showed significant increases in IL-6 levels compared to air controls (Figure 3B). In BALF, MCP-1 levels were unchanged in all exposed mice compared to air controls (Figure 2C). In lung homogenate, MCP-1 levels in combined data were significantly decreased in Apple, Strawberry, Wintergreen, and Smooth & Mild Tobacco exposed mice compared to air controls (Figure 3C). In all male mice exposure groups, a significant decrease in MCP-1 levels compared to air controls in lung homogenate was observed, whereas for female mice, MCP-1 levels were not impacted by the exposures (Figure 3C).

### 3.4. Alterations in Matrix Metalloproteinase Levels in Lungs by E-cigarette Flavors

**Figure 4:**
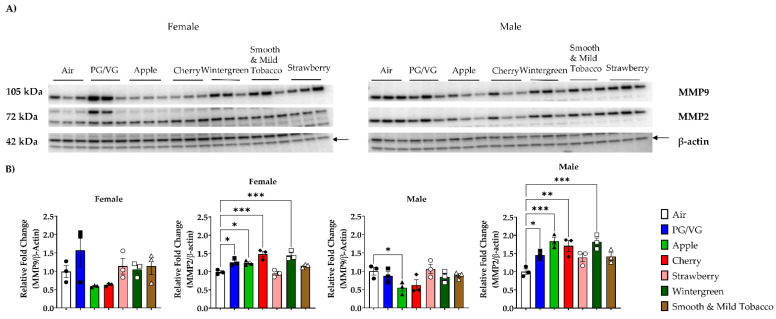
Effects of acute flavored e-cigarette exposure on matrix metalloprotease protein levels in lung homogenate. Mice were exposed to air, PG/VG, and e-liquid flavors “Apple”, “Cherry”, “Strawberry”, “Wintergreen”, and “Smooth & Mild Tobacco” for 3 days for 1 h per day. Mice were sacrificed twenty-four hours after the final exposure. Protein levels for matrix metalloproteinases were measured in lung homogenate using Western blot. (**A**) MMP2 and MMP9 protein abundance in mouse lung homogenate from male and female exposed mice. (**B**) Band intensity was measured using densitometry and data are shown as fold change compared to air control mice. Data are shown as mean ± SEM with individual data points represented by the following symbols: Air (black circles), PG/VG (black squares), Apple (black triangles), Cherry (black diamonds), Strawberry (white circles), Wintergreen (white squares), Smooth & Mild Tobacco (white triangles), with * *p* < 0.05, ** *p* < 0.01, and *** *p* < 0.001 vs. air controls. *n* = 3 for male- and female-only groups.

To determine the effect of flavored e-cigarettes on extracellular remodeling proteins, MMP protein levels were measured in lung homogenate. In all female mice exposure groups, there was no significant change in the relative fold change of MMP9 protein levels compared to air controls (Figure 4B, Figures S1–S3). Exposure to PG/VG, Apple, Cherry, and Wintergreen resulted in a significant increase in the relative fold change of MMP2 protein levels in female mice compared to air controls (Figure 4B, Figures S1–S3). Male mice exposed to Apple displayed a significant decrease in the relative fold change of MMP9 protein levels compared to air controls (Figure 4B, Figures S1–S3). In male mice exposed to PG/VG, Apple, Cherry, and Wintergreen resulted in a significant increase in the relative fold change of MMP2 protein levels compared to air controls (Figure 4B, Figures S1–S3).

## 4. Discussion

In this study, we investigated the immune-inflammatory effects of exposure to flavored e-cigarettes. To determine the potential inhalation effects of flavoring chemicals added into e-liquids, we determined the concentration of five distinct flavoring chemicals (maltol, ethyl maltol, benzaldehyde, methyl salicylate, and hexyl acetate), but the presence of other flavorants are still under investigation. Prior literature also indicates that these compounds have an abundant and widespread presence in market-available e-liquids [13,20,21]. While quantification of flavorants are important, a recently published study showed the inherent variability in lung deposition of flavoring chemicals as a function of inhalation modality: in “lung inhalers” nearly 100% retention of flavorants was observed, but lower retention was observed for “mouth inhalers” [22].

The chemicals found in these e-liquids (maltol, ethyl maltol, benzaldehyde, hexyl acetate, methyl salicylate, and hexyl acetate) are GRAS for ingestion, but the inhalation and respiratory effects of these chemicals are relatively understudied. Diacetyl, which has been used commercially as flavor additives in food for butter flavoring, is a flavoring chemical that has been found in e-liquids, but despite being GRAS for ingestion, the inhalation of this chemical has been found to result in the respiratory disease bronchiolitis obliterans, which showed the potential risk of inhaling these chemicals [23]. Although there are limited studies, one study has previously been conducted on the inhalation effects of benzaldehyde in Sprague-Dawley rats and found that exposure to 500, 750, and 1000 ppm benzaldehyde displayed dose-dependent increases in nasal irritation [24]. Previous preliminary data from our lab exposing C57 BL/6 J mice (*n* = 2–3) to PG/VG and benzaldehyde, following the same methodology described in thisstudy, has shown a potential trend of an increase in pro-inflammatory cytokine production in BALF, although the small sample size does not allow for the determination of significant changes (Figure S4).

Few studies have focused solely on the effect of flavors/flavoring chemicals in e-cigarette exposure, with most studies focusing on the effects of nicotine or the effects of the base components of e-liquids. One study that has investigated the respiratory effects of the flavoring chemical vanillin exposed C57 BL/6 J mice to 70:30 VG:PG with or without vanillin for 6 weeks. This study found similar results to the results herein, with no significant change in macrophages count and CD8 T cells from VG/PG with or without vanillin, while also finding a significant increase in CD4 T cells in mice exposed to VG/PG but also found an increase in VG/PG with vanillin contrary to the flavor exposure results herein [25]. Another study conducted on flavored e-cigarettes, in which C57 BL/6 J mice were exposed for two weeks to a menthol-flavored e-cigarette with 1.8% nicotine. Contrary to the results herein, macrophage cells were significantly increased in e-cigarette-exposed mice; meanwhile, no significant changes were found in neutrophil and lymphocyte cell counts such as the results herein [26]. One study conducted on the base component of e-liquids exposed female BALB/c to PG and VG alone. Similar to the results of our female mice exposed to PG/VG, PG and VG alone resulted in no significant change in total cell count and macrophage cell count compared to air controls, while contrary to the results herein, PG and VG alone did not result in a significant increase in neutrophil count [27]. In another study looking at the base component of e-liquids, female C57 BL/6 J mice were exposed to 60:40 PG:VG for four months. Comparable to the results herein, there was no significant change in macrophage cell counts in PG/VG; meanwhile, unlike the results herein, there was no significant change in neutrophil or lymphocyte cell counts in PG/VG exposures [28]. In another study conducted on exposure to PG for a three-day exposure, contrary to the results herein, exposure to PG resulted in a significant decrease in total cell counts and macrophage cell count, along with no significant change in neutrophil and CD4 T-cells [15]. A study conducted on the effects of PG for 1 month, similar to the results herein, found no significant change in total cell count, macrophage cell counts, or CD8 T-cells compared to air controls, while, differing from the results herein, there was no significant change in CD4 T-cells [29].

In line with the increase in IL-6 levels by PG/VG and Cherry exposures in lung homogenate, exposure of C57 BL/6 J mice to e-cigarette with 18 mg/mL nicotine found a significant increase in IL-6 RNA levels in the lung tissue [30]. However, exposure to 60:40 PG:VG in C57 BL/6 J female mice found no change in IL-6 levels in lung homogenate [28]. In line with the results from the Wintergreen flavor exposure, menthol-flavored C57 BL/6 J mouse exposure had no change in MCP-1 levels, although this exposure resulted in a significant decrease in IL-6 levels in BALF [26]. In contrast to results herein, another C57 BL/6 J e-cigarette exposure found that PG/VG did not alter IL-6 levels, but tobacco flavored exposure significantly increased IL-6 levels in lung homogenate [31]. In alternative mice strains, ENDS exposure in βENaC resulted in a significant increase in cytokines associated with lung fibrosis [32]. While exposure to PG/VG with nicotine in A/J mice resulted in a significant increase in RNA levels of cytokines associated with chronic obstructive pulmonary disease (COPD) [33].

The results of this study are one of the first evidence to show the sex-specific effects of nose-only exposure to e-cigarette flavors. The results herein found that male mice exposed to Smooth & Mild tobacco resulted in a significant increase in neutrophil count in BALF, while all e-cigarette exposures resulted in a significant decrease in MCP-1 levels in lung homogenate. In female mice exposed to Cherry, there was a significant increase in levels of KC and IL-6 in lung homogenate, while in female mice exposed to Apple, there was a significant increase in neutrophil count in BALF. In PG/VG-exposed female mice, there was a significant increase in neutrophils in BALF and a significant increase in KC levels in lung homogenate. There are limited current studies that have investigated the sex-specific effects of e-cigarette exposure. One study investigating the effects of PG and PG with nicotine on C57 BL/6 J mice in a sex-specific manner found that female mice exposed to PG with nicotine had a significant increase in neutrophil and CD8 T-cell counts, while male mice exposed to PG with nicotine were found to have a significant increase in lung inflammatory cytokines [15]. A study conducted on cigarette smoke exposure on spontaneously hypertensive rats found that male mice had significant increases in macrophage cell counts and tumor necrosis factor-alpha levels showing a male-specific increase in inflammation contrary to the results herein but similar to the results of Wang et al. [34].

Alterations in MMP2 and MMP9 levels due to e-cigarette exposures are important, since both MMP2 and MMP9 gelatinolytic activity have been found to be increased in the sputum in both asthmatic and COPD patients [35]. Comparable to the results herein, exposure of C57 BL/6 J mice to PG found that there was no change in MMP9 levels in exposed mice [15]. However, e-cigarette exposures to PG/VG with nicotine resulted in an increase in MMP9 and other lung protease levels [33]. Cell studies have found that alveolar macrophages and neutrophils treated with e-cigarette condensate significantly increased MMP9 [36,37]. MMP9 levels have also been found to be elevated in the plasma and bronchoalveolar lavage in e-cigarette users [38,39]. Consistent with our MMP2 results, increased MMP2 levels have also been found in mice exposed to PG and increased MMP2 levels in the bronchoalveolar lavage of chronic e-cigarette users [15,29,39]. Although the effects of e-cigarette exposures on cytokine/chemokine levels, MMP levels, and BALF cell counts have different effects in this and other studies, these differences may come down to the methodology for e-cigarette exposures. Each study utilizes different devices and puffing profiles for mouse exposures, along with different e-liquids with differences in nicotine concentration, flavors, and the ratio of PG and VG. These differences between studies demonstrate the need for a standardized methodology for mouse exposures to reduce potential differences between studies and allow for greater comparisons between studies.

## 5. Conclusions

Based on the results in this study, flavored e-cigarettes showed both increases in lung inflammation and resolution. Mice exposed to PG/VG, Cherry, and Smooth & Mild Tobacco resulted in an increase in lung inflammation due to the increases in KC and IL-6 levels in lung homogenate along with infiltration of neutrophils in BALF. These exposures may also have sex-specific alterations, with Smooth & Mild Tobacco exposure only resulting in a significant increase in neutrophil cell counts in male mice. Meanwhile, in Cherry exposure, KC and IL-6 levels were increased in lung homogenate only in female mice. In PG/VG exposures, only female mice had a significant increase in neutrophil cell count and a significant increase in KC levels in lung homogenate. Despite the increases in inflammatory cytokines in Cherry and PG/VG, the increases in MMP2 levels potentially indicate that these exposures have begun to shift away from inflammation and towards tissue repair and resolution. In contrast, other exposures, such as Wintergreen flavor, resulted in a decrease in lung inflammation, with a decrease in MCP-1 levels and increases in MMP2 levels. Further studies are in progress to determine the chronic exposures to flavored e-cigarettes on long-term pulmonary effects and the potential sex-specific effects. This study revealed that flavor-based e-cigarette exposure elicited sex-specific alterations in lung inflammation, with cherry flavors/benzaldehyde eliciting female-specific and tobacco flavor resulting in male-specific increases in lung inflammation. This highlights the toxicity of flavored chemicals and the further need for the regulation of flavoring chemicals.

## Figures and Tables

**Table 1:** Flavoring chemical and propylene glycol and vegetable glycerin quantification in e-liquids. E-liquids were analyzed by ^1^H NMR using a Bruker Advance 500 MHz NMR spectrometer with 128 scans with a 4.7 s repetition rate and a 30° flip angle, with 64 k data points. Flavoring chemical concentrations and propylene glycol and vegetable glycerin quantification were representatives of the average of the three samples ± SEM.

E-liquids	Flavoring Chemicals	Concentration	PG:VG
Apple	Hexyl Acetate	0.43 ± 0.04 mg/mL	46:54
	Ethyl Maltol	0.30 ± 0.05 mg/mL	
Cherry	Benzaldehyde	0.12 ± 0.01 mg/mL	51:49
Strawberry	Ethyl Maltol	0.32 ± 0.05 mg/mL	50:50
	Maltol	0.24 ± 0.04 mg/mL	
Wintergreen	Methyl Salicylate	9.70 ± 0.50 mg/mL	49:51
Smooth & Mild Tobacco	Maltol	1.13 ± 0.02 mg/mL	49:51

## Data Availability

Not applicable.

## Supplementary Materials

The following supporting information can be downloaded at: https://www.mdpi.com/article/10.3390/toxics10080471/s1, Figure S1: Full image of MMP2 for male and female exposed mice; Figure S2: Full image of MMP9 for male and female exposed mice; Figure S3: Full image of β-actin for male and female exposed mice; Figure S4: Alterations in pro-inflammatory cytokines/chemokine release in bronchoalveolar lavage fluids due to acute exposure to PG/VG and PG/VG with benzaldehyde.
