# Correction: Lamb et al. Nose-Only Exposure to Cherry- and Tobacco-Flavored E-Cigarettes Induced Lung Inflammation in Mice in a Sex-Dependent Manner. *Toxics* 2022, *10*, 471

**DOI:** 10.3390/toxics11100845

**Published:** 2023-10-08

**Authors:** Thomas Lamb, Thivanka Muthumalage, Jiries Meehan-Atrash, Irfan Rahman

**Affiliations:** Department of Environmental Medicine, School of Medicine & Dentistry, University of Rochester Medical Center, Rochester, NY 14620, USA; thomas_lambjr@urmc.rochester.edu (T.L.); thivanka_muthumalage@urmc.rochester.edu (T.M.); jiries.m.a@gmail.com (J.M.-A.)

In the original publication [1], there was an error in Figure 4 as published. There was an inadvertent replication of male MMP9 bands in place of male MMP2 during the preparation of this figure, although the corrected respective images of the full western blots were published in the supplemental material Figures S1 and S2. The corrected Figure 4 appears below. The densitometry graphs remain unchanged since the values were generated with the correct full-blot images. The authors state that the scientific conclusions are unaffected. This correction was approved by the Academic Editor. The original publication has also been updated.

## Figures and Tables

**Figure 4:**
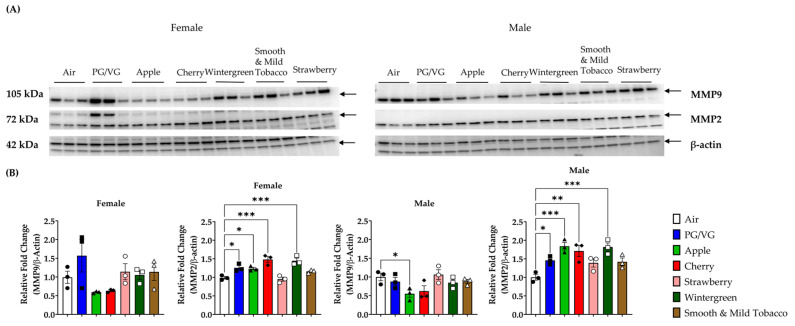
Effects of acute flavored e-cigarette exposure on matrix metalloprotease protein levels in lung homogenate. Mice were exposed to air, PG/VG, and e-liquid flavors “Apple”, “Cherry”, “Strawberry”, “Wintergreen”, and “Smooth & Mild Tobacco” for 3 days for 1 h per day. Mice were sacrificed twenty-four hours after the final exposure. Protein levels for matrix metalloproteinases were measured in lung homogenate using Western blot. (**A**) MMP2 and MMP9 protein abundance in mouse lung homogenate from male and female exposed mice. (**B**) Band intensity was measured using densitometry and data are shown as fold change compared to air control mice. Data are shown as mean ± SEM with * *p* < 0.05, ** *p* < 0.01, and *** *p* < 0.001 vs air controls. *n* = 3 for male- and female-only groups.
