# Supplementary material for: Nose-Only Exposure to Cherry- and Tobacco-Flavored E-Cigarettes Induced Lung Inflammation in Mice in a Sex-Dependent Manner
Source: Toxics. 2022 Aug 13;10(8):471. doi: 10.3390/toxics10080471 (PMC9413458; doi:10.3390/toxics10080471)
Supplement: Supplementary file 1 [file toxics-10-00471-s001.zip › toxics-1838696-supplementary.pdf]

toxics

MDPI

Supplementary Materials: Nose-only Exposure to Cherry and Tobacco Flavored E-cigarettes Induced Lung Inflammation in Mice in a Sex-dependent Manner

Thomas Lamb, Thivanka Muthumalage, Jiries Meehan-Atrash, and Irfan Rahman

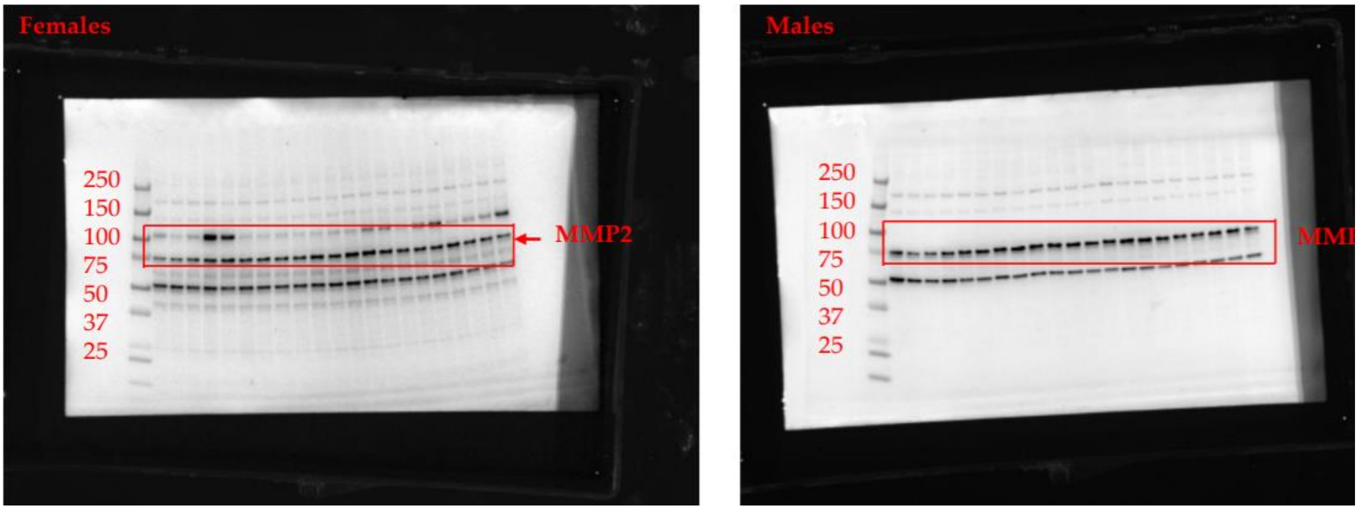

Figure S1. Full Image of MMP2 for Male and Female Exposed Mice. Images represent the full blot for MMP2 with the female mice blot representing the blot after being stripped for MMP9 and reprobed with MMP2 (dilution 1:1000) and with the male mice blot representing the initial probe with MMP2 (dilution 1:1000)

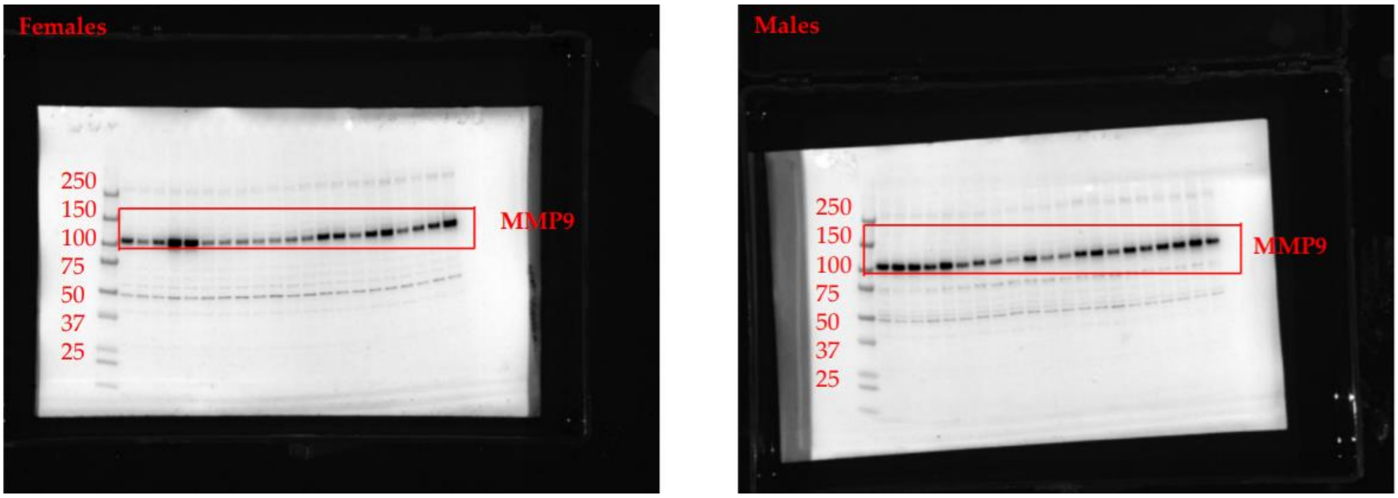

Figure S2. Full Image of MMP9 for Male and Female Exposed Mice

Images represent the full blot for MMP9 with the male mice blot representing the blot after being stripped for MMP2 and re-probed with MMP9 (dilution 1:1000) and with the female mice blot representing the probe with MMP9 (dilution 1:1000)

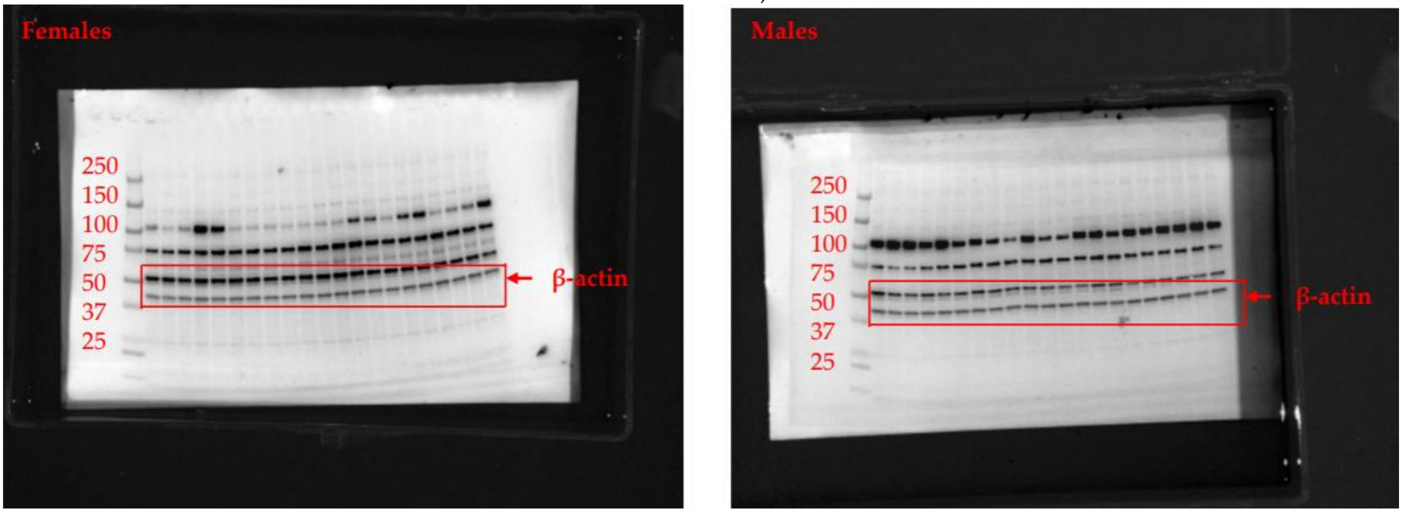

Figure S3. Full Image of β-actin for Male and Female Exposed Mice Images represent the full blot for β-actin with both blots representing the image after each membrane has been stripped and finally probed with β-actin (dilution 1:2000)

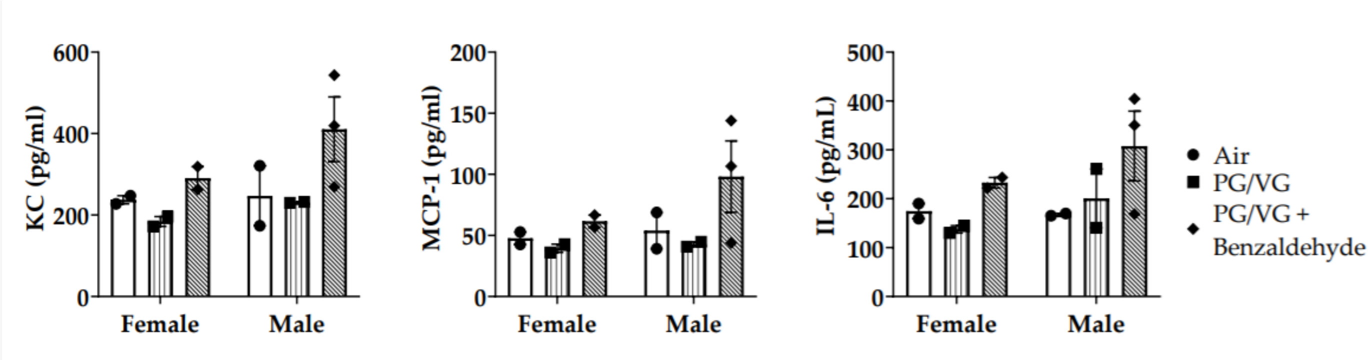

Figure S4. Alterations in pro-inflammatory cytokines/chemokine release in bronchoalveolar lavage fluids due. C57BL6/J mice aged, 13–15 weeks old, were exposed for three days to air, 50:50 PG/VG, and 50:50 PG/VG with 280 µg/mL for one hour per day. Mice were exposed to a puffing profile utilized to expose mice was set at two puffs per minute at an inter-puff interval of thirty seconds, with a three-second puff duration and a puff volume of 51 ml. Mice were sacrificed 24-hours post final exposure and were lavaged 3 times with 0.6 ml of 0.05% fetal bovine serum in 0.9% NaCl. BALF was then run for pro-inflammatory cytokines/chemokines KC, MCP-1, and IL-6. Data are shown as mean ± SEM, n = 2–3.
